# The role of patient and surgeon characteristics on the treatment decision for displaced midshaft clavicle fractures in athletes: a global survey

**DOI:** 10.1016/j.xrrt.2025.100662

**Published:** 2026-01-31

**Authors:** Koen M. Plug, Melle M. Broekman, David Ring, Lukas P.E. Verweij, Michel P.J. van den Bekerom, Jelle P. van der List, Science of Variation Group, A.J.H. Vochteloo, A. Prkic, B.F. Hearon, C. Casstevens, C. Zalavras, C. Garnavos, C. Medina, C.L. Moreno, D.C. Wascher, D. London, E.J. Harvey, E.P. Hofmeister, F. Frihagen; J.M. Patino, K. Rumball, K. Kabir, J.H. Marsh, L.K. Cannada, L.M.S.J. Poelhekke, L.W. van der Plaat, M.J. Richard, M.M.E. Wijffels, M. Quell, M. Swiontkowski, M.W. Grafe, M. Tyllianakis, M. Shafi, N. Akabudike, N. Parnes, N. Schep, R. Llombart, R.A. Schaefer, R. Buckley, R. Dunbar, R.E. Van Demark, R.S. Gilbert, S. Campbell, S. Farr, S.L. Henry, S.A. Kennedy, T. Schepers, T. Havlicek, T.M. McLaurin, T.S. Guitton, T. Siff, V. Giordano, W.J. Willems

**Affiliations:** a Department of Orthopedic Surgery, Amsterdam UMC, University of Amsterdam, Amsterdam Movement Sciences, Amsterdam, The Netherlands; b Amsterdam Shoulder and Elbow Center of Expertise (ASECE), Amsterdam, The Netherlands; c Dell Medical School—The University of Texas at Austin, Austin, TX, USA; d Faculty of Behavioral and Movement Sciences, Department of Human Movement Sciences, Vrije Universiteit Amsterdam, Amsterdam Movement Sciences, Amsterdam, The Netherlands; e Academic Center for Evidence-based Sports medicine (ACES), Amsterdam UMC, Amsterdam, The Netherlands; f Amsterdam Collaboration for Health and Safety in Sports (ACHSS), International Olympic Committee (IOC) Research Center, Amsterdam UMC, Amsterdam, The Netherlands; g Shoulder and Elbow Unit, Department of Orthopedic Surgery, OLVG, Amsterdam, The Netherlands; h Department of Orthopaedic Surgery and Sports Medicine, The Ohio State University Wexner Medical Center, Jameson Crane Sports Medicine Institute, Columbus, OH, USA

**Keywords:** Clavicle fractures, Athletes, Surgical decision-making, Surgeon characteristics, Patient characteristics, Shared decision-making return to sport, Treatment variation

## Abstract

**Background:**

Diaphyseal clavicle fractures are common in athletes. While surgery can reduce the risk of nonunion and expedite return to sport, it carries risks and may lead to hardware removal. How patient and surgeon characteristics influence treatment decisions in athletes with displaced midshaft clavicle fractures has not been clearly defined.

**Methods:**

Participants from the Science of Variation Group were invited to review randomized radiographs of displaced midshaft clavicle fractures paired with different patient scenarios that included age, sports level, dominant arm, type of sport, timing within the season, presence of an upcoming significant sporting event occurring within the next three months, and five distinct sets of radiographs. Surgeons rated their likelihood of offering surgery (scale: 0-100). Multivariable analysis identified factors associated with the likelihood of offering surgery. Data are presented as regression coefficients (RCs) with corresponding 95% confidence intervals (CIs). Statistical significance was set at *P* < .05. Analyses were conducted in STATA version 13.

**Results:**

A total of 94 surgeons reviewed 470 different clinical scenarios. Surgeons were primarily based in the United States (43%) and Europe (32%); 90% were male and 82% were involved in teaching trainees. The predominant specialty was orthopedic trauma surgery (40%), followed by hand/wrist surgery (28%) and shoulder/elbow surgery (14%). Compared with weekend athletes, surgeons were significantly more likely to offer surgery to professional athletes (RC = 14; CI = 7.8-20; *P* < .001), corresponding to a mean increase of 14 points on the 0-100 scale. The presence of an important upcoming sporting event was also associated with a higher likelihood of offering surgery (RC = 9.5; CI = 4.5-15; *P* < .001), corresponding to a mean increase of 9.5 points on the 0-100 scale. Male surgeons were more likely to offer surgery than female surgeons (RC = 16; CI = 4.1-26; *P* = .004), corresponding to 16 points higher on the 0-100 scale. This observed association should be interpreted with caution, given the limited number of female respondents.

**Conclusions:**

Surgical decision-making for displaced midshaft clavicle fractures in athletes is influenced not only by anatomical considerations but also by contextual factors directly related to functional recovery. Overall, the findings suggest that surgeons weigh functional demands and timing heavily when making these decisions, underscoring the importance of shared decision-making tailored to the individual athlete.

Clavicle fractures are common fractures with an estimated incidence of 29-64 per 100,000.^28^^,^^30^^,^^36^ Approximately 30% of clavicle fractures occur during sports, and most fractures involve the diaphysis.^9^^,^^33^^,^^36^ These fractures can be either treated with nonoperative or operative treatment. With nonoperative treatment, healing depends on secondary healing and union rates range from 84% to 100%^22^^,^^40^^,^^46^^,^^48^^,^^49^^,^^51^ with symptomatic malunion from 14% to 43%.^5^^,^^22^^,^^39^^,^^40^^,^^45^ Operative treatment has been shown to lead to union in 95%-98%^6^^,^^14^^,^^27^^,^^35^^,^^40^^,^^47^^,^^49^^,^^51^ but carries the risk for complications in 3%-31%.^14^^,^^20^^,^^24^^,^^25^ In addition, up to 25% of patients require implant removal due to discomfort or failure.^14^^,^^24^ Long-term functional outcomes appear comparable between operative and nonoperative treatment.^49^ In athletes, operative treatment has been shown to lead to higher return-to-sport rates and quicker return-to-sport times, with return occurring at approximately 9 weeks after surgery compared with about 21 weeks after nonoperative treatment, corresponding to an average 12-week earlier return.^35^

Many factors determine the decision for nonoperative vs. operative treatment. These include, but are not limited to, patient characteristics, sports activities, patient preference, and cultural environment with a large variety between countries and hospitals.^26^^,^^41^^,^^50^ In addition to these considerations, surgeons' decisions may also be influenced by implicit bias, defined as unconscious cognitive tendencies that can influence clinical judgment. In athletes, several studies have focused on return-to-sport rates and times, union rates, and predictors of nonunion.^2^^,^^5^^,^^6^^,^^10^^,^^35^ One of the most under-recognized drivers for this decision is the surgeon preference on the treatment of clavicle fractures in athletes, but studies have been limited assessing how implicit bias may affect decision-making in athletes with clavicle fractures.

Therefore, the goal of this study is to assess the role of surgeon preference on the treatment of clavicle fractures in athletes and the factors that drive surgical decision-making. Previous studies on distal radius, proximal humerus, and terrible triad fractures have been shown valuable in providing insights in what drives surgeons in decision-making, but this has not been done for clavicle fractures.^16^^,^^23^^,^^32^ The hypothesis is that the patient characteristics young age and professional sports level are associated with recommending surgery, along with surgeon characteristics of young age and trauma subspecialty. Given the small sizes of several surgeon subgroups, analyses regarding surgeon characteristics should be considered exploratory.

## Methods

### Study design and setting

A survey was sent to surgeon members of the Science of Variation Group (SOVG), which is an international collaborative of orthopedic, fracture, and plastic surgeons that studies sources of variation in health care in May 2022. Members of SOVG do not receive financial incentives for participation but do receive group authorship or acknowledgment. Following the initial invitation, two weekly reminders were sent. SurveyMonkey (Palo Alto, CA, USA) was used.

### Response variables

Surgeons were asked to review five patient scenarios, each containing randomized radiographs, personal, and situational factors. Participants were asked to assess the likelihood of offering operative treatment for a displaced midshaft clavicle fracture, expressed on a scale from 0 (“I would not offer surgery”) to 100 (“I would strongly encourage surgery”). A Likert scale was chosen instead of a binary outcome (surgery vs. no surgery) because treatment recommendations in clinical practice are rarely absolute. This approach is consistent with methodological evidence showing that Likert-type scales are appropriate for capturing variation in clinical judgment.^29^

### Explanatory variables

Each participant assessed five hypothetical case scenarios of adults who participate in sports with a displaced midshaft clavicle fracture. These scenarios were generated by randomizing the following factors: (1) age, (2) sports level, (3) dominant arm, (4) type of sport, (5), timing within the season, (6) presence of an upcoming significant sporting event, and (7) five distinct sets of radiographs (Table I). Each factor had a limited number of options. Age was categorized as 18, 24, 30, and 36 years. Sports level was classified as weekend athlete (participates recreationally), high-level amateur (trains and competes), or professional (earns a living by competing). Dominant arm was recorded as either yes or no. The type of sport was classified into four categories: noncontact, nonoverhead sport; noncontact, overhead sport; contact nonoverhead sport; and contact, overhead sport. Timing within the season was designated as either pre-/mid-season or end of the season. The presence of an important upcoming sports event occurring within the next three months was noted as yes or no. The final randomized factor included five sets of diaphyseal clavicle-fracture radiographs. All radiographs were standardized anteroposterior clavicle views acquired under our institution's routine imaging protocol. Image quality and anatomical clarity were verified before inclusion. These fractures were classified according to Robinson classification and represented displaced fracture patterns, with at least three showing >100% displacement, three showing significant shortening, two demonstrating complete loss of bony contact, and two exhibiting clear comminution. Specifically, they were classified as type 2A2 (n = 2), type 2B1 (n = 1), and type 2B2 (n = 2) (Supplementary Appendix S1).

**Table I:** Patient characteristics.

Variables	Value∗
N cases	470
Age (yr)	
18	Equal probability assigned
24	
30	
36	
Sports level	
Weekend athlete	Equal probability assigned
High-level amateur	
Professional	
Dominant arm involved	
Yes	50% (randomized)
No	50% (randomized)
Type of sport	
Noncontact, nonoverhead	Equal probability assigned
Noncontact, overhead	
Contact, nonoverhead	
Contact, overhead	
Season timing	
Pre-season/mid-season	50% (randomized)
End of season	50% (randomized)
Upcoming significant event	
Yes	50% (randomized)
No	50% (randomized)
Fracture type (Robinson)	
2A2	20% (n = 1 case per 5)
2B1	40% (n = 2 cases per 5)
2B2	40% (n = 2 cases per 5)

∗ Values were randomized with equal probability across predefined categories. Percentages are theoretical based on random assignment across the 470 total hypothetical cases (5 per participant). No stratification or weighting was applied.

### Patient characteristics

Hypothetical case characteristics were randomly assigned from predefined categories with equal probability, resulting in an approximately balanced distribution across factors such as age, sports level, dominant arm, type of sport, timing within the season, presence of an upcoming significant sporting event, and fracture type. A sample survey and the corresponding five randomized cases, including the radiographs as presented to the reviewers, are available in Supplementary Appendix S1.

The secondary study question examined whether surgeon characteristics were associated with the likelihood of offering surgery. These characteristics included gender, age, continent of practice, total years of surgical experience, years of experience managing clavicle fractures, involvement in supervising trainees, and surgical specialty.

### Statistical analysis

Descriptive statistics were conducted for all participants. Then, a mixed multilevel linear regression analysis was employed to identify patient-related factors associated with surgeon-rated likelihood to offer surgery. In addition, a linear regression analysis was performed to study surgeon characteristics associated with the surgeon-rated likelihood of offering surgery.

Regression coefficients (RCs) were calculated to quantify the strength of these associations, along with their corresponding 95% confidence intervals (CIs). A positive RC indicates an increased likelihood of offering surgery, while a negative RC signifies a decreased likelihood. Statistical significance was determined at a *P* value threshold of less than .05. All analyses were performed using STATA MP version 13 (Stata Statistical Software Release 13 2013; StataCorp, College Station, TX, USA.).

### Sample size calculation

An a priori sample size calculation indicated that 131 observations would be necessary to achieve 80% statistical power based on a linear regression model explaining 25% of the variance in the rate of surgery.^8^^,^^13^ This is consistent with effect sizes reported in prior SOVG scenario-based studies.^4^^,^^31^ This calculation assumed that the tested factors would increase the likelihood of recommending surgery by 50%, with a baseline surgery rate of 10% and alpha set at 0.05. Given that each surgeon evaluates 5 cases, an initial requirement of 27 surgeons was established to provide adequate statistical power. To account for the correlation of observations within individual surgeons (due to nesting), the sample size was subsequently doubled, resulting in a target of 54 surgeons in total.^11^ G∗ Power version 3.1^13^ was used for the sample size calculations.

## Results

### Surgeon characteristics

A total of 133 surgeons among a cohort of approximately 200 who typically participate in at least one SOVG survey per year completed the survey (66.5%). Twenty-four respondents indicated they were not familiar with the treatment of midshaft clavicle fractures and were therefore excluded. An additional 13 responses were excluded due to incompleteness, resulting in a final sample of 94 eligible participants.

The participating surgeons who considered this survey relevant to their clinical practice were primarily based in the United States (43%) and Europe (32%), with additional respondents from Canada, Asia, Australia, and other regions. Most participants were male (90%), and the majority were involved in teaching trainees (82%). The most common subspecialty was orthopedic trauma surgery (40%), followed by hand or wrist surgery (28%), and shoulder and elbow surgery (14%). Participants had been in practice for mean 14 years, with 43% having practiced less than ten years. Each surgeon treated a mean 10 midshaft clavicle fractures annually (Table II).

**Table II:** Demographics of surgeons.

Variables	Value∗
N	94
Male	90% (85)
Continent	
US	43% (40)
Europe	32% (30)
Other	26% (24)
Years of practice	
0-5	23% (22)
6-10	19% (18)
11-20	30% (28)
21-30	28% (26)
Supervising	82% (77)
Subspecialty	
Ortho trauma	40% (38)
Hand/wrist	28% (26)
Shoulder/elbow	14% (13)
Other	18% (17)
Midshaft clavicle fractures/yr	10 (5-30)

∗ Value is displayed as median with interquartile range for continuous nonparametric variables, as mean with standard deviation for continuous variables with normal distribution, and as number with percentage for categorical variables.

### Patient factors associated with an increased likelihood of offering surgery

Patient factors associated with a greater likelihood of offering surgical treatment included sports level and the presence of an important upcoming event. Compared with weekend athletes (reference category), professional athletes received on average 14-point higher scores on the 0-100 recommendation scale (RC = 14 [CI = 7.8-20], *P* < .001) (Table III). In addition, cases with a significant sporting event within three months were rated 9.5 points higher than otherwise similar cases without such an event (RC = 9.5 [CI = 4.5-15], *P* < .001) (Table III). No significant associations were identified regarding age, dominant arm, type of sport, timing within the season, or the set of radiographs (Table III).

**Table III:** Mixed multilevel linear regression analysis of patient factors associated with likelihood to offer surgery.

	Regression coefficient (95% confidence interval)	Standard error	*P* value	Δ Akaike
Age				−5.6
18	*Reference value*			
24	−0.58 (−7.6 to 6.4)	3.6	.87	
30	−1.9 (−8.7 to 5.0)	3.5	.59	
36	−2.1 (−9.0 to 4.9)	3.5	.56	
Level of sports				18
Weekend athlete	*Reference value*			
High-level amateur	3.5 (−2.7 to 9.6)	3.2	.27	
Professional	14 (7.8-20)	3.0	**<.001**	
Dominant arm affected				−1.9
No	*Reference value*			
Yes	0.36 (−4.7 to 5.4)	2.6	.89	
Type of fracture				−4.0
1	*Reference value*			
2	4.3 (−2.9 to 11)	3.6	.24	
3	−0.048 (−7.2 to 7.1)	3.7	.99	
4	−1.4 (−8.6 to 5.7)	3.6	.70	
5	−2.5 (−9.7 to 4.7)	3.7	.49	
Type of sport				−2.2
No contact, overhead	*Reference value*			
No contact, no overhead	3.6 (−3.2 to 11)	3.5	.30	
Contact, overhead	5.6 (−1.1 to 12)	3.4	.10	
Contact, no overhead	6.0 (−0.97 to 13)	3.6	.092	
Timing in season				0.6
Ending	*Reference value*			
Beginning/mid	4.2 (−0.81 to 9.2)	2.5	.10	
Important event coming up				12
No	*Reference value*			
Yes	9.5 (4.5-15)	2.6	**<.001**	

Bold indicates statistical significance, *P* < .05.

### Surgeon factors associated with an increased likelihood of offering surgery

Surgeon factors associated with a greater likelihood of offering surgical treatment included male gender (RC = 16 [CI = 4.1-26], *P* = .004) (Table IV). In absolute terms, this corresponds to an increase of 16 on the 0-100 scale for males compared with females. Although the overall sample size was sufficient according to the a priori calculation, the number of female surgeons was very limited. Therefore, the observed association between male gender and surgical recommendation should be interpreted with caution. However, no significant associations were identified regarding age, continent of practice, total years of surgical experience, years of experience managing clavicle fractures, involvement in supervising trainees, or surgical specialty (Table IV).

**Table IV:** Linear regression analysis of surgeon factors associated with likelihood to offer surgery.

	Regression coefficient (95% confidence interval)	Standard error	*P* value	Partial R^2^
Gender				
Female	*Reference value*			
Male	16 (4.1-26)	5.3	**.004**	0.019
Continent				
US	*Reference value*			
Europe	−6.3 (−14 to 1.7)	4.1	.12	0.0054
Other	3.9 (−4.7 to 13)	4.4	.37	0.0018
Years of practice				
0-5	*Reference value*			
6-10	0.24 (−9.7 to 10)	5.1	.96	0.0000049
11-20	5.3 (−3.7 to 14)	4.6	.25	0.0029
21-30	−2.1 (−11 to 6.9)	4.6	.65	0.00047
Supervising				
No	*Reference value*			
Yes	−4.0 (−13 to 4.6)	4.4	.37	0.0019
Subspecialty				
Hand/wrist	*Reference value*			
Shoulder/elbow	−0.80 (−11 to 9.9)	5.4	.88	0.000049
Ortho/trauma	6.5 (−1.8 to 15)	4.2	.12	0.0053
Other	9.6 (−1.3 to 20)	5.5	.085	0.0067
Clavicle fractures treated	0.054 (−0.12 to 0.23)	0.091	.55	0.00079

Bold indicates statistical significance, *P* < .05.

## Discussion

The most important findings of this study were that professional-level sports participation and an upcoming important sports event occurring within the next three months were key predictors for surgeons recommending surgery. The initial hypothesis was only partly confirmed. The observed tendency of male surgeons to recommend surgery should be interpreted cautiously given the small number of female respondents in our cohort. These results suggest that surgical decision-making is influenced not only by anatomical considerations, but also by contextual factors that are directly related to functional recovery. Professional athletes and important upcoming sporting events determine the urgency of return to activity, which is consistent with evidence that operative fixation can expedite functional recovery in athletes, with prior literature reporting that return to sport may occur approximately 12 weeks earlier compared with nonoperative management.^35^

Operative treatment of displaced midshaft clavicle fractures lowers the risk of nonunion and can expedite recovery.^14^^,^^19^^,^^27^^,^^35^^,^^37^^,^^40^^,^^51^ Therefore, surgery is often preferred in athletes and young, active individuals.^35^^,^^37^ However, it carries surgical risks, including implant removal, infection, and sensory disturbances.^6^^,^^14^^,^^20^^,^^24^^,^^25^

Our finding that surgeons more frequently recommended surgery for professional athletes and to those with an important upcoming event highlights an important ethical and clinical consideration. If operative fixation is perceived as offering advantages such as faster recovery, its selective use in these athletes may result in unequal access to potential benefits. Conversely, if the surgical risks are considered substantial, the greater exposure of the athletes to these risks indicates that surgeons may accept these risks more readily in this group. This pattern may be explained by a different risk–reward ratio: professional athletes not only tend to return to sport earlier,^18^ but also face significant financial and career-related consequences, earning less after injury^38^ and impacting team performance and revenue.^12^^,^^17^ Taken together, this could mean that athletes and nonathletes do not face the same opportunities or risks when surgery is being considered.

The variation in decision-making based on patient factors aligns with previous research on treatment variation for midshaft clavicle fractures.^50^ Notably, fracture characteristics were not associated with surgical decisions, despite some types (eg, 2B2) carrying a higher risk of nonunion without surgery.^2^ This suggests that functional and contextual demands can outweigh anatomical risk factors in certain populations, particularly elite athletes. Other studies also highlight the influence of patient age, social factors, medical history, and overall health on treatment decisions.^50^ Given that the radiographs presented in the fictitious cases were identical to the routine clinical radiographic views used for clavicle fractures, this pattern likely reflects real-world decision-making rather than a limitation of the study design.

The findings have implications for shared decision-making, regardless of competition level. Surgeons and patients may prioritize fast return to sport but must also weigh surgical risks such as implant removal, infection, and sensory disturbances.^6^^,^^24^ Importantly, nonoperative treatment often yields comparable long-term outcomes.^47^^,^^49^ Misconceptions regarding treatment options are common and should be addressed with sensitivity.^42^^,^^43^ Shared decision-making is crucial in clavicle fracture management,^44^ with many patients preferring physician involvement and valuing shared input.^26^ The perceived quality of this process strongly predicts satisfaction.^44^ Even though long-term outcomes are comparable, operative treatment tends to yield higher satisfaction, emphasizing the importance of individualized care.^44^^,^^47^

Surgical decisions also vary by surgeon characteristics. Shoulder specialists are more likely to recommend surgery,^1^ and regional differences affect recommendations.^10^ Surgeons are more likely to offer surgery to patients than choose it for themselves and show more confidence in self-treatment decisions.^21^ Less experienced orthopedic surgeons tend to favor surgery.^1^ Differences between studies may also arise due to variation in physician beliefs about surgical indications, the extent to which patient preferences are incorporated, and differences in training backgrounds or health care systems. In addition, regional practice incentives such as bundled payments,^34^ reimbursement structures,^15^ and varying malpractice climates^7^ may further shape surgical tendencies and could partly explain variation observed across previous studies. Finally, surgeon-specific factors such as implicit bias or local financial and structural incentives may contribute to these discrepancies.^3^

This study has limitations. First, most SOVG members are male academics, and only a subset participated, limiting generalizability, especially regarding surgeon gender. Yet, recognizing implicit bias remains important. Second, fictitious cases may not fully reflect clinical complexity. Third, only seven patient factors were included, omitting others such as physical attributes, mental health, socioeconomic status, and environmental influences. Finally, each variable had limited preset options, which may oversimplify clinical nuance. Still, the design likely captures relevant decision-making trends. Furthermore, the study did not include patient-reported outcome measures or objective functional recovery data, which limits the ability to validate whether surgeons' decision-making recommendations align with actual patient outcomes.

## Conclusion

This study shows that surgical decisions for displaced midshaft clavicle fractures in athletes are influenced by both medical and contextual factors. Professional-level sports participation and the presence of an important sporting event occurring within the next three months were the strongest patient characteristics associated with recommending surgery. Male gender was the only surgeon-related factor, a finding that should be interpreted cautiously given the limited number of female respondents. These results suggest that treatment recommendations are guided by the balance between the risk of complications and the functional demands, underscoring the importance of shared decision-making to align treatment with individual goals. Future studies should link surgeons' decision-making patterns to actual functional and patient-reported outcomes in athletes to determine whether these contextual influences ultimately improve or compromise long-term results.

## Acknowledgment

The authors would like to thank all of the following members of the Science Of Variation Group who participated in their study: A.J.H. Vochteloo (Centre for Orthopaedic Surgery and Sports Medicine, OCON, Hengelo, The Netherlands), A. Prkic (Department of Orthopedic Surgery, Northwest Clinics, Alkmaar, The Netherlands), B.F. Hearon (University of Kansas School of Medicine in Wichita, Wichita, KS, USA), C. Casstevens (Division of Hand, Upper Extremity, and Microvascular Surgery, Department of Orthopaedic Surgery, University of California, San Diego (UCSD), La Jolla, CA, USA), C. Zalavras (Department of Orthopaedics, Keck School of Medicine, University of Southern California, LAC+USC Medical Center, Los Angeles, CA, USA), C. Garnavos (Division of Hand, Upper Extremity, and Microvascular Surgery, Department of Orthopaedic Surgery, University of California, San Diego (UCSD), La Jolla, CA, USA), C. Medina (Hospital Universidad del Norte, Soledad, Atlántico, Colombia), C.L. Moreno (Hand and Microsurgery Division, Department of Orthopedics and Traumatology, Hospital Universitario Fundación Santa Fe de Bogotá, Bogotá, Colombia; School of Medicine, Universidad de los Andes, Bogotá, Colombia), D.C. Wascher (Department of Orthopaedic Surgery, University of New Mexico, Albuquerque, NM, USA), D. London (Department of Orthopaedic Surgery, University of Missouri, Columbia, MO, USA), E.J. Harvey (Faculty of Medicine and Health Sciences, McGill University, Montreal, Quebec, Canada; Division of Orthopaedic Surgery, McGill University, Montreal, Quebec, Canada), E.P. Hofmeister (Department of Orthopaedic Surgery Naval Medical Center San Diego, San Diego, CA, USA), F. Frihagen (University of Oslo, Oslo, Norway), J.M. Patino (Hospital Militar Central, Orthopaedic and Traumatology, Buenos Aires, Argentina), K. Rumball (Emplify Health La Crosse Orthopaedics), K. Kabir (Helios University Hospital Wuppertal, Wuppertal, Germany), L. Marsh (University of Iowa Healthcare: Iowa City, IA, USA), L.K. Cannada (Novant Health Perry & Cook Orthopedics & Sports Medicine, Charlotte, NC, USA), L.M.S.J. Poelhekke (Department of Surgery, Maasziekenhuis Pantein, Beugen, The Netherlands), L.W. van der Plaat (Department of Traumatology and Orthopedic Surgery, St.-Antonius-Hospital Kleve, Klinik für Unfallchirurgie und Orthopädie, Kleve, Germany), M.J. Richard (Duke University Hospital, Durham, NC, USA), M.M.E. Wijffels (Trauma Research Unit, Department of Surgery, Erasmus MC, University Medical Center Rotterdam, Rotterdam, The Netherlands), M. Quell (Wiener Krankenanstaltenverbund, Vienna, Austria), M. Swiontkowski (University of Minnesota, department of Orthopaedic Surgery, Minneapolis, MN, USA), M.W. Grafe (Redwood Orthopaedic Surgery Associates), M. Tyllianakis (Orthopaedic Department, Patras University Hospital, Patras, Greece), M. Shafi (Phoenix Hospital, Abu Dhabi, Orthopaedics Surgery, United Arab Emirates), N. Akabudike (Department of Orthopaedics, University of Maryland School of Medicine, Baltimore, MD, USA), N. Parnes (Carthage Area Hospital, Carthage, MO, USA), N. Schep (University of Amsterdam, Department of Surgery, Amsterdam, The Netherlands), R. Llombart (Orthopedic Surgery Department, University Clinic of Navarra, Pamplona, Spain), R.A. Schaefer (Department of Orthopaedic Surgery, The Johns Hopkins University, Baltimore, MD, USA), R. Buckley (Department of Orthopaedic Surgery, UC Davis School of Medicine, Sacramento, CA, USA), R. Dunbar (University of Washington Department of Orthopaedics), R.E. Van Demark (Sanford USD Medical Center), R.S. Gilbert (Dell Medical School, Austin, TX, USA), S. Campbell (Department of Orthopaedic Surgery, UC Davis School of Medicine, Sacramento, CA, USA), S. Farr (Abteilung für Kinderorthopädie und Fußchirurgie, Orthopädisches Spital Speising, Wien, Österreich, Vienna, Austria; Vienna Bone and Growth Center, European Reference Network on Rare Bone Diseases Full Member, Wien, Österreich, Vienna, Austria), S.L. Henry (Central Texas, Inc., Austin, TX, USA), S.A. Kennedy (University of Washington, Seattle, WA, USA), T. Schepers (Amsterdam UMC, Trauma Unit, Meibergdreef, The Netherlands), T. Havlicek (Ljubljana University Medical Centre, Ljubljana, Slovenia), T.M. McLaurin (NYU Langone Medical Center, Orthopedic Surgery Department, New York City, NY, USA), T.S. Guitton (Department of Plastic Surgery, University Medical Center Groningen, Groningen, The Netherlands), T. Siff (Dell Medical School, Austin, TX, USA), V. Giordano (Hospital Municipal Miguel Couto, Rio de Janeiro, RJ, Brazil), W.J. Willems (Lairesse Kliniek, Orthopaedic department, Amsterdam, The Netherlands).

## Disclaimers

Funding: No funding was disclosed by the authors.

Conflicts of interest: David Ring MD PhD reports other items from Skeletal Dynamics; personal fees as Deputy Editor for Clinical Orthopedics and Related Research; personal fees from Universities and Hospitals; personal fees from Lawyers; personal fees from Health Services and Resource Administration and Department of Justice; personal fees from Premier Healthcare Solutions; personal fees from Wolters Kluwer Health; and grants from National. Erie P. Hofmeister MD receives infrequent honorarium for teaching (not within the past year) with/for Trimed Inc. Frede Frihagen has received support for educational activities from DePuy Synthes and Smith&Nephew. Lisa K. Cannada is a participant author in AAOS CPG on Clavicle Fractures. Marc Richard discloses consulting, royalties, and speakers bureau from Acumed; consulting from Bioventus; consulting and speakers bureau from DJO; consulting and speakers bureau from Fielding Orthopedics; and speakers bureau from Medartis. The other authors, their immediate families, and any research foundation with which they are affiliated have not received any financial payments or other benefits from any commercial entity related to the subject of this article.
