# Supplementary material for: The role of patient and surgeon characteristics on the treatment decision for displaced midshaft clavicle fractures in athletes: a global survey
Source: JSES Rev Rep Tech. 2026 Jan 31;6(2):100662. doi: 10.1016/j.xrrt.2025.100662 (PMC12993187; doi:10.1016/j.xrrt.2025.100662)
Supplement: Supplementary Appendix S1 [file mmc1.docx]

**Case 1**

A 18 year old high level amateur athlete who trains several times a week and competes comes to your office.

He/she has a displaced midshaft clavicle fracture on his/her dominant arm.

He/she plays a no contact, no overhead sport.

It is the beginning/middle of a sporting season.

He/she has an extra important upcoming sports event 10 weeks after his/her first visit to you, in which he/she really wants to participate.

**Radiograph case 1**


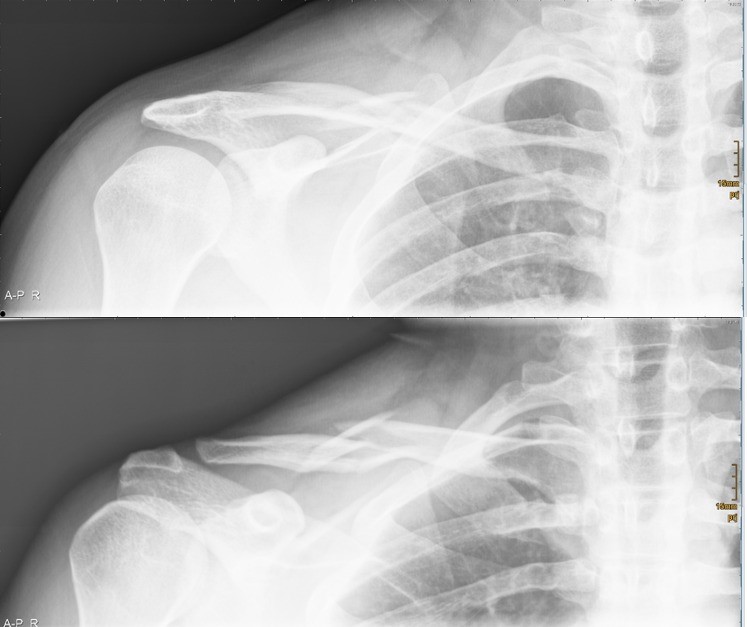


**How likely are you to  offer surgery?**

| 0 (I would not offer surgery) |  | 10 (I would strongly encourage surgery) |
| --- | --- | --- |

| 1 | 2 | 3 | 4 | 5 | 6 | 7 | 8 | 9 | 10 |
| --- | --- | --- | --- | --- | --- | --- | --- | --- | --- |

**Case 2**

A 24 year old weekend athlete who plays sports for health and enjoyment comes to your office.

He/she has a displaced midshaft clavicle fracture on his/her dominant arm.

He/she plays a contact, no overhead sport

It is the end of a sporting season.

He/she has no extra important upcoming sports event 10 weeks after his/her first visit to you.

**Radiograph case 2**


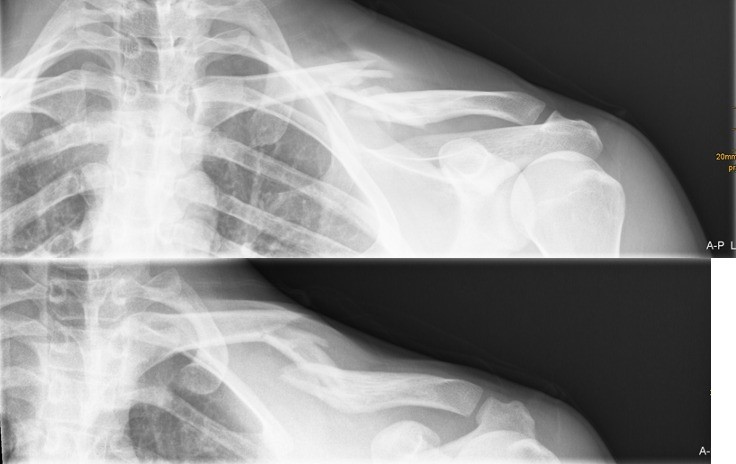


**How likely are you to  offer surgery?**

| 0 (I would not offer surgery) |  | 10 (I would strongly encourage surgery) |
| --- | --- | --- |

| 1 | 2 | 3 | 4 | 5 | 6 | 7 | 8 | 9 | 10 |
| --- | --- | --- | --- | --- | --- | --- | --- | --- | --- |

**Case 3**

A 30 year old high level amateur athlete who trains several times a week and competes comes to your office.

He/she has a displaced midshaft clavicle fracture on his/her dominant arm.

He/she plays a no contact, overhead sport

It is the end of a sporting season.

He/she has no extra important upcoming sports event 10 weeks after his/her first visit to you.

**Radiograph case 3**


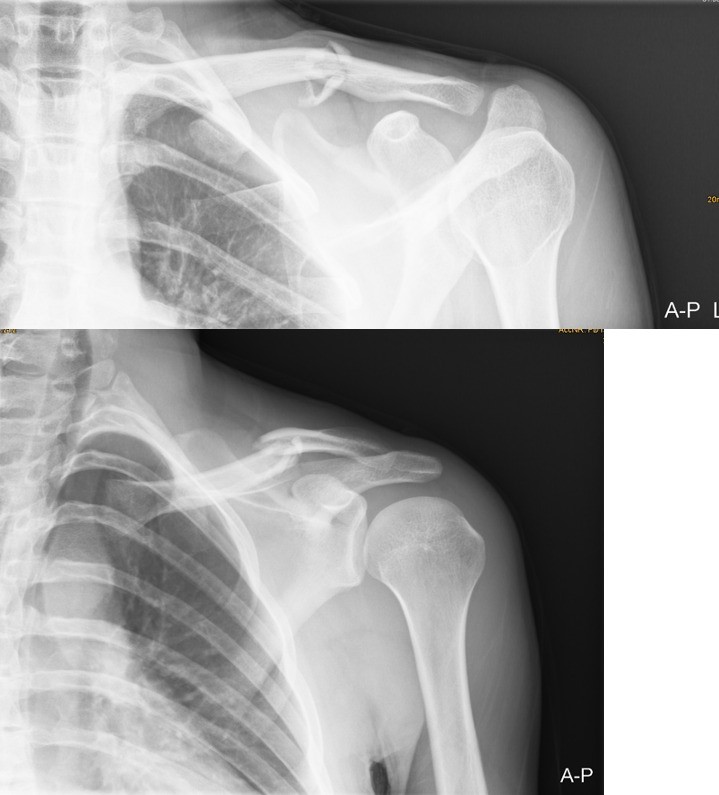


**How likely are you to  offer surgery?**

| 0 (I would not offer surgery) |  | 10 (I would strongly encourage surgery) |
| --- | --- | --- |

| 1 | 2 | 3 | 4 | 5 | 6 | 7 | 8 | 9 | 10 |
| --- | --- | --- | --- | --- | --- | --- | --- | --- | --- |

**Case 4**

A 24 year old professional athlete who earns a living by competing in a sport comes to your office.

He/she has a displaced midshaft clavicle fracture on his/her dominant arm.

He/she plays a no contact, no overhead sport

It is the end of a sporting season.

He/she has an extra important upcoming sports event 10 weeks after his/her first visit to you, in which he/she really wants to participate.

**Radiograph case 4**


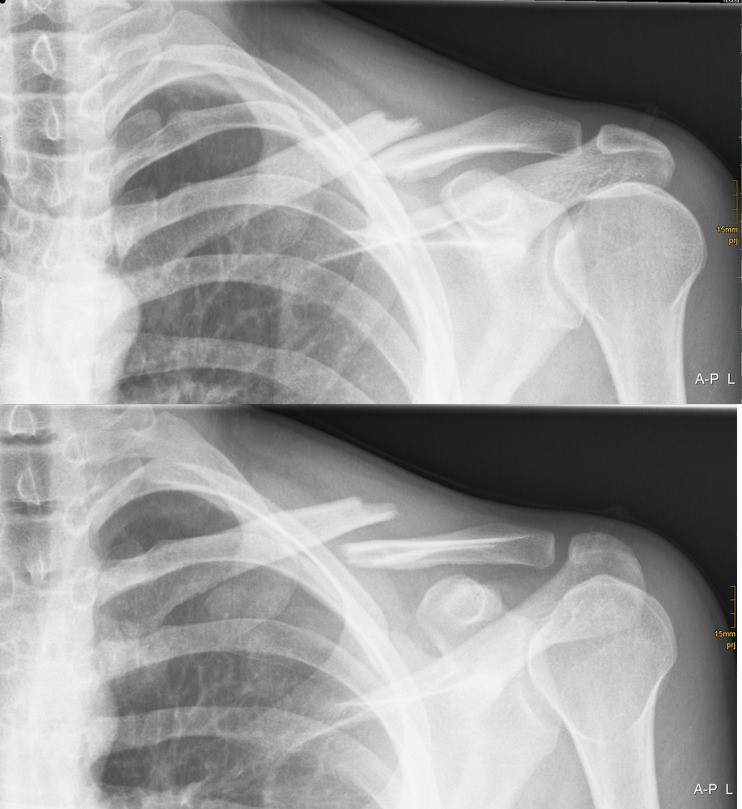


**How likely are you to  offer surgery?**

| 0 (I would not offer surgery) |  | 10 (I would strongly encourage surgery) |
| --- | --- | --- |

| 1 | 2 | 3 | 4 | 5 | 6 | 7 | 8 | 9 | 10 |
| --- | --- | --- | --- | --- | --- | --- | --- | --- | --- |

**Case 5**

A 36 year old high level amateur athlete who trains several times a week and competes comes to your office.

He/she has a displaced midshaft clavicle fracture on his/her non-dominant arm.

He/she plays a contact, overhead sport

It is the beginning/middle of a sporting season.

He/she has no extra important upcoming sports event 10 weeks after his/her first visit to you.

**Radiograph case 5**


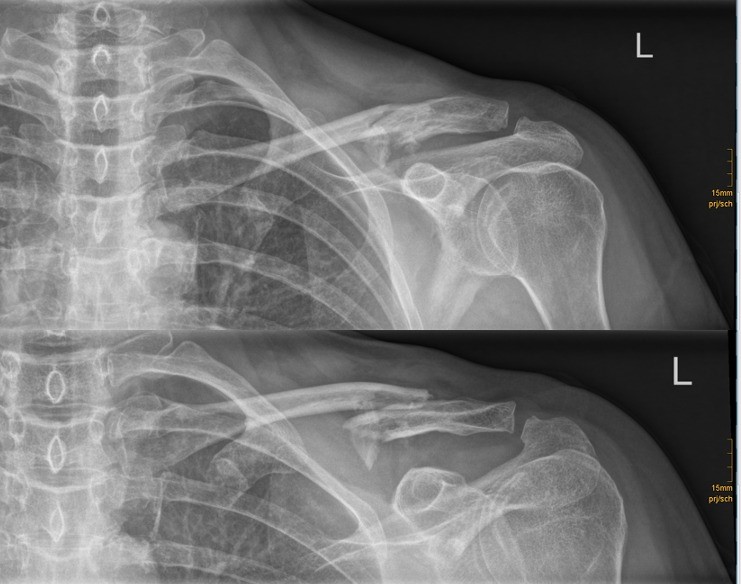


**How likely are you to  offer surgery?**

| 0 (I would not offer surgery) |  | 10 (I would strongly encourage surgery) |
| --- | --- | --- |
|  |  |  |

| 1 | 2 | 3 | 4 | 5 | 6 | 7 | 8 | 9 | 10 |
| --- | --- | --- | --- | --- | --- | --- | --- | --- | --- |
